# Estimating the population exposed to a risk factor over a time window: A microsimulation modelling approach from the WHO/ILO Joint Estimates of the Work-related Burden of Disease and Injury

**DOI:** 10.1371/journal.pone.0278507

**Published:** 2022-12-30

**Authors:** Bálint Náfrádi, Hannah Kiiver, Subas Neupane, Natalie C. Momen, Kai N. Streicher, Frank Pega

**Affiliations:** 1 Labour Administration, Labour Inspection and Occupational Safety and Health Branch, International Labour Organization, Geneva, Switzerland; 2 Eurostat, Luxembourg, Luxembourg; 3 Department of Climate Change, Environment and Health, World Health Organization, Geneva, Switzerland; Universidade Federal de Minas Gerais, BRAZIL

## Abstract

**Objectives:**

Burden of disease estimation commonly requires estimates of the population exposed to a risk factor *over a time window* (year_t_ to year_t+n_). We present a microsimulation modelling approach for producing such estimates and apply it to calculate the population exposed to long working hours for one country (Italy).

**Methods:**

We developed a three-model approach: Model 1, a multilevel model, estimates exposure to the risk factor at the first year of the time window (year_t_). Model 2, a regression model, estimates transition probabilities between exposure categories during the time window (year_t_ to year_t+n_). Model 3, a microsimulation model, estimates the exposed population over the time window, using the Monte Carlo method. The microsimulation is carried out in three steps: (a) a representative synthetic population is initiated in the first year of the time window using prevalence estimates from Model 1, (b) the exposed population is simulated over the time window using the transition probabilities from Model 2; and (c) the population is censored for deaths during the time window.

**Results:**

We estimated the population exposed to long working hours (i.e. 41–48, 49–54 and ≥55 hours/week) over a 10-year time window (2002–11) in Italy. We populated all three models with official data from Labour Force Surveys, United Nations population estimates and World Health Organization life tables. Estimates were produced of populations exposed over the time window, disaggregated by sex and 5-year age group.

**Conclusions:**

Our modelling approach for estimating the population exposed to a risk factor over a time window is simple, versatile, and flexible. It however requires longitudinal exposure data and Model 3 (the microsimulation model) is stochastic. The approach can improve accuracy and transparency in exposure and burden of disease estimations. To improve the approach, a logical next step is changing Model 3 to a deterministic microsimulation method, such as modelling of microflows.

## Introduction

**Fig 1:**
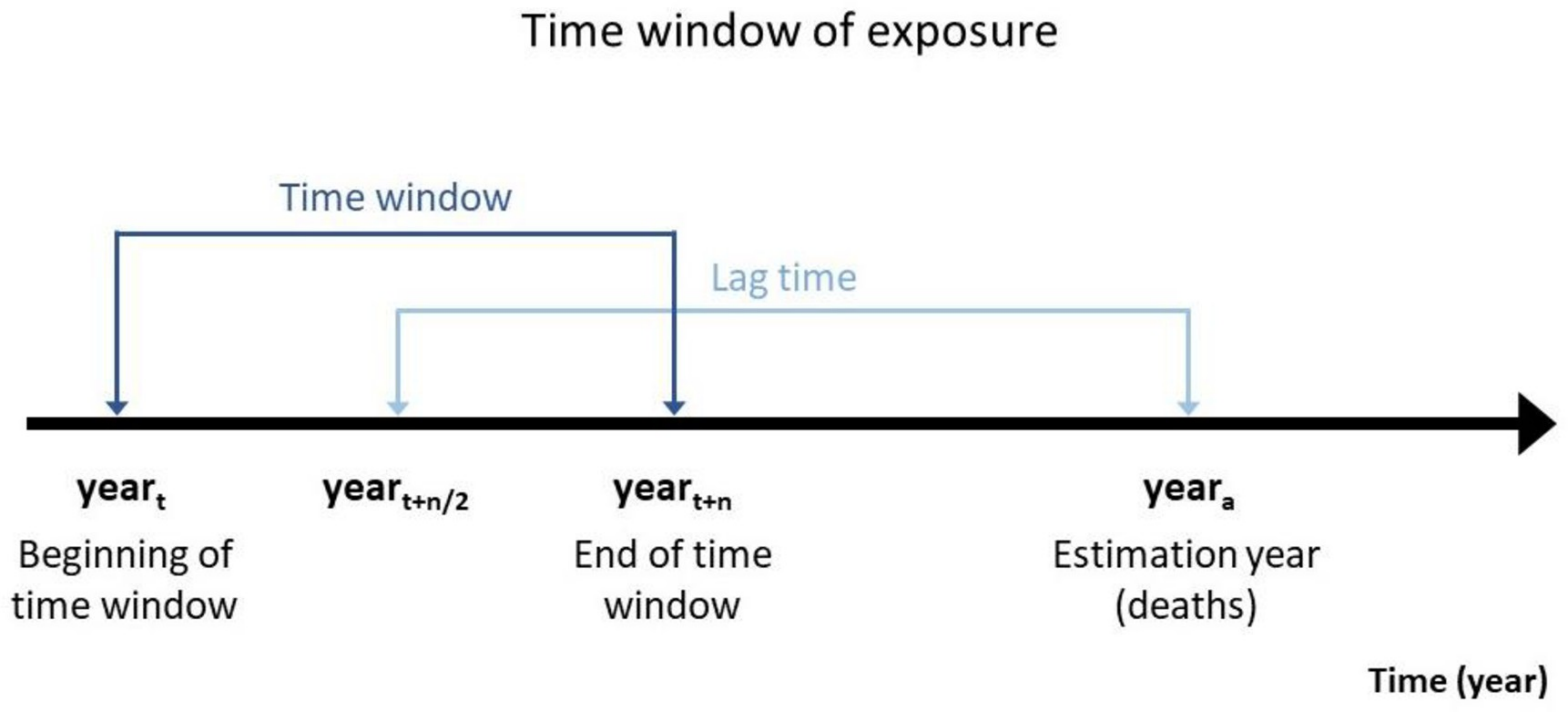
Definition of time window. We seek to estimate the burden of disease (such as the number of deaths or disability-adjusted live years lost) at *year*_*a*_ that is attributable to past exposure to a risk factor. For this, we require estimates of the number of persons exposed to an exposure category over the time window (*year*_*t*_ to *year*_*t*+*n*_). The known or assumed lag time is *year*_*t*+*n*/2_ to *year*_*a*_ and we place the time window around the lag year. We then seek to estimate the number of persons exposed to the category *during* the time window.

The World Health Organization (WHO) and the International Labour Organization (ILO), supported by a large number of individual experts [1–20], produce estimates of exposure to selected occupational risk factors and, consecutively, of the work-related burden of disease attributable to these exposures (i.e. the burden of disease that can be attributed to exposure to a particular risk factor in the past) [21–24]. These estimates are called the WHO/ILO Joint Estimates of the Work-related Burden of Disease and Injury (WHO/ILO Joint Estimates; see https://www.who.int/teams/environment-climate-change-and-health/monitoring/who-ilo-joint-estimates). A crucial step when producing the WHO/ILO Joint Estimates, as with estimating other burden of disease attributable to exposure to a risk factor, is estimating the prevalence of exposure to this risk factor *over a time window* (Fig 1). A time window is defined as the “period in which an exposure can have adverse or protective effects on development and subsequent disease outcome” [25]. Improved accuracy in estimating exposure prevalence leads to more accurate burden of disease estimation. WHO and ILO have developed tools and approaches to use in systematic reviews of prevalence of exposure [26–28]. In this article, we describe a novel modelling approach from the WHO/ILO Joint Estimates, which can be used for producing estimates for all burden of disease studies, but here, we focus on work-related (or occupational) burden of disease where possible. The approach was developed after WHO and ILO identified the need for improved modelling of exposure over time for the production of official estimates of exposure to occupational risk factors, taking into consideration transitions between exposure categories over time.

Two main different methods exist for estimating prevalence of exposure over a time window (S2 Box). First, the Global Burden of Disease Study used occupation as a proxy for exposure to some occupational risk factors [29–32]. The prevalence of exposure to an occupational risk factor in the lag year (according to the lag time i.e. the time period between exposure to the risk factor and the occurrence of the health outcome) was multiplied by a constant factor of 4, the occupational turnover (OT; i.e. the ratio of the prevalence of a category of exposure to the risk factor at one year divided by the prevalence of the same exposure category over a time window), to estimate the prevalence of exposure over a time window. This OT rate did not vary over time, nor by cohort defined by sex and/or age group. Longitudinal data are not used.

Second, in the United Kingdom Burden of Cancers Study, staff turnover factors were estimated using new starters in years in the time window [33,34]. This method estimated starters in the past year as a proportion of the average number of people employed. It presented turnover rates of 4 and 6 for short-latency (20 years) and long-latency (40 years) risk exposure periods, respectively, meaning that prevalence of exposure in a year taking into account turnover is estimated to be 4 times larger (for short latency) and 6 times larger (for long latency) than the point estimate of the exposure in the same year. This method still makes limited use of longitudinal data.

In this article, we propose a new method to estimate the prevalence of exposure to an occupational risk factor over a time window, as input data for burden of disease estimates. This method makes more use of longitudinal data to model the movement of the target population between exposure categories. We describe the methods using an example with three exposure categories (41–48, 49–54, and ≥55 hours/week) for the occupational risk factor of exposure to long working hours over a 10-year window for Italy, using data from the European Union-Labour Force Survey (EU-LFS). We discuss the implications of our findings in relation to previous methods, including the gain in precision it provides, as well as next steps to further improve our approach.

## Materials and methods

**Fig 2:**
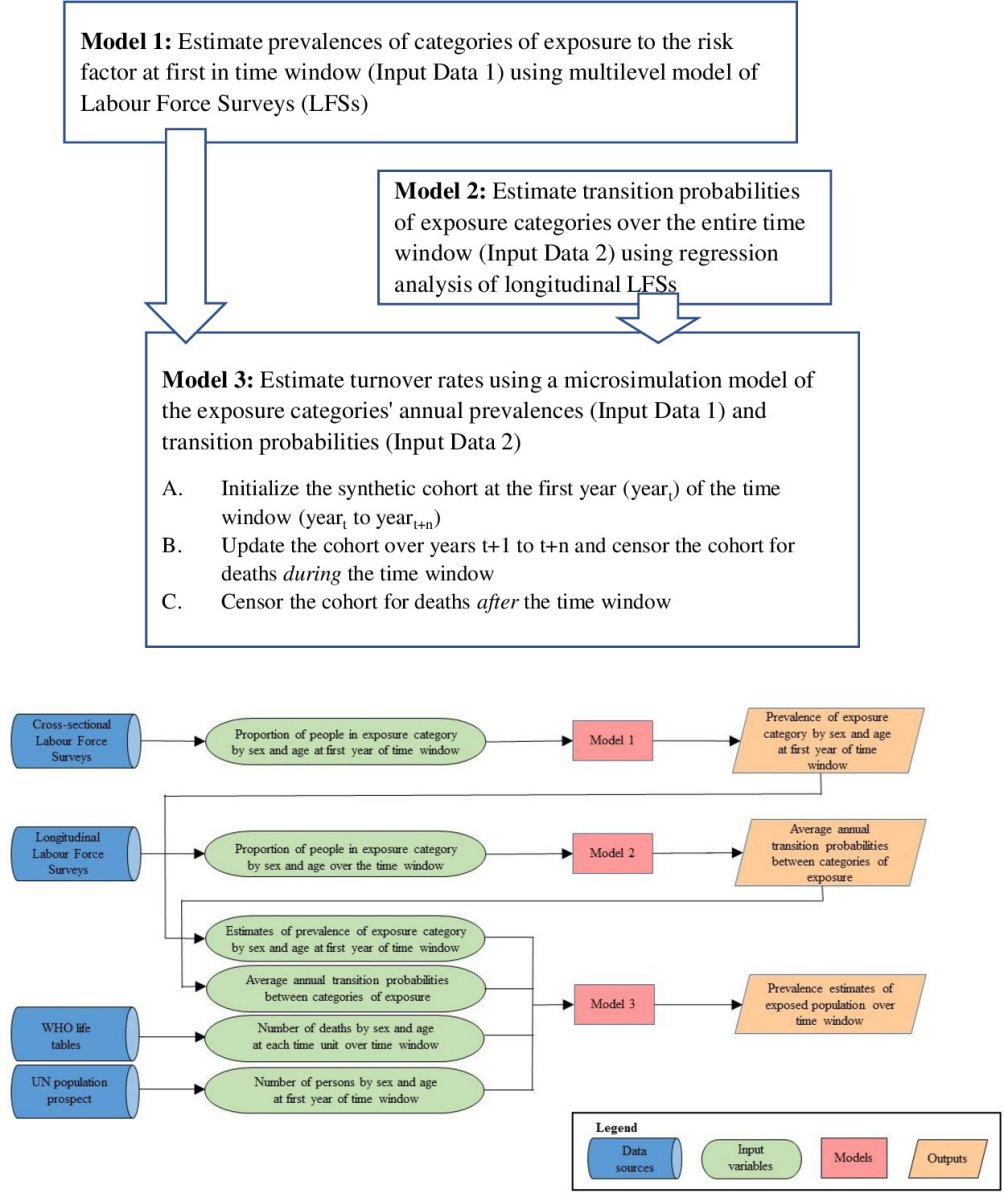
Flow diagram of the data sources and modelling approach. **a)** Overview of the steps of the method to estimate prevalence of exposure to an occupational risk factor over a time window. **b)** Flow diagram of the data sources and modelling approach.

We developed a modeling approach based on three consecutive models. We produced two sets of estimates (Models 1 and 2 in Fig 2) as input data for the microsimulation model. First, the prevalence of exposure to an occupational risk factor was estimated as input data at the first year of the time window, *year*_*t*_ (Model 1). Second, we modeled transition probabilities (i.e. the probability of changing the level of exposure between the exposure categories) for exposure categories during the time window, *year*_*t*_ to *year*_*t*+*n*_ (Model 2). Third, we developed a Monte Carlo method (MCM) based microsimulation model to estimate the exposed population over the time window based on these transition probabilities (Model 3). The methods and the data sources used in each of three models are described in detail below.

### Model 1: Estimate of prevalence of exposure to the risk factor for the first year in time window

We estimated the prevalence of risk factor exposure for each exposure category for the first year in the time window using an established multilevel model that predicted the prevalence over time and geographic location [35]. The multilevel model is of the form:

$$proportion_{i}=A_{i}(sex,age,country)+B_{i}(sex,age,country)\times year$$

where *proportion*_*i*_ is the proportion of the population in the exposure category *i*; *year* is the survey year; and *sex*, *age* and *country* are the sex, age group and country of the population. The intercept *A*_*i*_ (*sex*, *age*, *country*) and slope *B*_*i*_ (*sex*, *age*, *country*) of the year dependence of *proportion*_*i*_ are calculated with a multilevel model, with sex and age group as fixed effects, and sex and age group as random effects, nested in the country. (Note that we modelled age group as a categorical variable, not a continuous variable as the database we used for this model only included age group categories.) We assumed that residuals follow a normal distribution (see S3 Table for R code).

### Model 2: Estimate of transition probabilities of exposure categories over the time window

We estimated average transition probabilities between exposure categories by running a weighted multinomial logit regression model including sex and age as a fractional polynomial as regressors, using a matched sample of longitudinal data. From the coefficients estimated, we derived predicted probabilities of transitioning between exposure categories by cohorts defined by sex and age group. Including age as a continuous variable borrowed strength from the distribution of age to estimate age groups with limited numbers of observations. Thus, the final estimates, particularly for the highest and lowest age groups, are to some extent driven by the choice of function. This methodology builds on the approach developed by Kiiver & Espelage [36]. The multinomial logit regression model is of the form:

$$probability_{j}=\frac{e^{\beta_{j}X_{j}}}{1+\sum_{\alpha }e^{\beta_{\alpha }X_{j}}}$$

where *β*_*j*_ is the set of regression coefficients describing the longitudinal weights associated with the transition *j*; *X*_*j*_ is a set of explanatory variables (sex and age as a fractional polynomial with maximal permitted degree of 2 associated with the transition *j*); and the summation (index*α*) goes through all possible transitions *j* (except the transition from *i* = 0 in *year*_*t*_ to *i* = 0 in *year*_*t*+1_, which was chosen as a pivot outcome).

Including age as a continuous variable allows us to borrow strength from the distribution of age to estimate age groups with limited numbers of observations. Thus, particularly for the highest and lowest age groups, the final estimates are to some extent driven by the choice of function. This methodology builds on the approach developed by Kiiver and Espelage [36] (in the cross sectional data we model, however, age group categories are pre-defined).

### Model 3: Estimate of the exposed population over the time window using a microsimulation model

Each individual in the synthetic population possesses a set of attributes, which may be updated at discrete time steps. In particular, individuals are often defined as belonging to one of a finite number of mutually exclusive and collectively exhaustive states, and events of interest are modelled as transitions from one state to another that occur according to a set of deterministic and/or stochastic rules governed by transition probabilities. To derive *proportion*_*k*_ for exposure category *k*, the microsimulation takes the following form:

$$proportion_{k}=\frac{1\sum_{l=1\ldots n}\delta_{k,max(S_{l})}}{n}$$

where the summation runs through individuals “alive” in the estimation year; *δ*_*k*_ is the Kronecker delta function; *S*_*l*_ is the sequence of all exposure categories the *l*^*th*^ individual was assigned to in each year in the time window; and where *max* denotes that the highest exposure category that the *l*^*th*^ individual experiences in the sequence.

We produced estimates of the number of exposed population over the time window in three steps (A-C Fig 2, Model 3), which are detailed below.

### Step A: Initialize the synthetic cohort at the first year (year_t_) of the time window (year_t_ to year_t+n_)

We initialized the synthetic cohort at the first year (*year*_*t*_) of the time window. The individuals in this cohort were set to be representative of the distribution of sex, age and the exposure category in the general population for the country. We determined the size of the synthetic cohort, and its sex and age composition, using the size, sex and age distributions from the UN estimates for the national population [37]. The initial exposure distribution is obtained from the estimated prevalence of the exposure categories (an output from the multilevel model, Model 1).

### Step B: Update the cohort over year_t+1_ to year_t+n_ and censor the cohort for deaths during the time window

Mutually exclusive and collectively exhaustive state of exposure category bands are defined during simulation; as individuals are simulated over time, each year the individual’s age is increased by 1-year steps. Following the principles of MCM, at each step, the exposure category band is reassigned stochastically based on exposure category *h*_*i*_, transition probabilities *h*_*i*,*j*_, and a computer-generated uniform random number *p* from (0–1) (all unique to the individual): the (0–1) interval was split into six parts defined by $\sum_{j=0}^{k}h_{i,j}$ at *k* = 0..5 and compared with *p*. Exposure category *h*_*i*_ was changed to the matching *h*_*k*_. At each year step, the alive state was consistently reassigned for each individual in a similar manner.

### Step C: Censor the cohort for deaths after the time window

At each year-step, the alive state was reassigned for each individual in a similar manner as the exposure category detailed in the previous paragraph. Here, however, the state of alive individuals only changed to dead if *p* was smaller than the probability of death, defined by sex and the actual age and year extracted from WHO standard life tables [38]. Those who survived until after the time window (i.e. to *year*_*a*_) formed the synthetic cohort and were further analyzed.

### Assessing the sensitivity of the modelling approach

The sensitivity of the modelling approach was tested by calculating the median relative error estimates for various input data uncertainty terms and their combinations using tornado plots.

## Example

We applied the modelling approach to estimate the population exposed to long working hours (i.e. 41–48, 49–54 and ≥55 hours/week) in time window 2002–2011 in Italy. The analysis was restricted to people aged 15 years and older. We used data from three sources that are publicly accessible: the United Nations population estimates (2002–2011 [37]), experimental longitudinal estimates from EU-LFS Italy for the years 2010/11 to 2017/18, and WHO life tables [39].

### Data

The cross-sectional data for Model 1 were derived from the Italian EU-LFS, for the years 1983 to 2018. A detailed description of the cross-sectional EU LFS is provided elsewhere [40]. The bands of exposure to working hour *h*_0_−*h*_5_ are *i* = 0: labour-force inactive; 1: 0–34 hours/week; 2: 35–40 hours/week; 3: 41–48 hours/week; 4: 49–54 hours/week; 5: ≥55 hours/week. S1 Table presents data sources, processes and steps of input variables.

The transition probability estimates are based on pseudo-longitudinal, reweighted EU-LFS microdata for Italy for the years 2010/11 to 2017/18 that are currently not accessible publicly. For rules governing access to anonymized EU-LFS microdata, see https://ec.europa.eu/eurostat/web/microdata [41]. The analysis was restricted to people aged 15 years and older. The pseudo-longitudinal data were derived from the Italian EU-LFS by matching the data from the annually overlapping samples, averaging over quarters per year, for the years 2010/2011 to 2017/2018. In the absence of personal identifiers, matching was based on household number, household sequence number, sex, and year of birth [42]. This pseudo-longitudinal sample was reweighted to match target year margins for labour market status of the cross-sectional data, by sex and 10-year age group. Summary statistics on the input data can be found in the respective references and in S2 Table.

### Models

Using Model 1, we estimated the prevalence of exposure at the first year of time window (*year*_*t*_ = 2002). All cohorts aged ≥15 years (as defined above) were followed. Estimates were calculated for 34 such cohorts, defined by sex (female, male) and 5-year age group (17 categories: 15–19, 20–24, …, 90–94, ≥95 years), e.g., women aged 15–19 years. In this article, for simplicity and comprehension, we present estimates for cohorts defined by sex only. The computer software R was used to produce exposure prevalence for the first year in time window (S3 Table for R code). In the microsimulation model (Model 3), these estimates were used as the first set of input data (Input Data 1).

Model 2, a regression model, was used to estimate transition probabilities between the six categories of exposure *h*_0_…*h*_5_ (as defined in the data section). Fractional polynomials with automated model selection in STATA 14 were used to model age for each separate regression run for the six exposure categories (i.e., *h*_0_…*h*_5_). The transition between these bands is captured through 36 transition probabilities, 30 of which are independent. A single set of transition probabilities per cohort is used for all years in the time window (*year*_*t*_ = 2000 to *year*_*t*+*n*_ = 2011).

**Fig 3:**
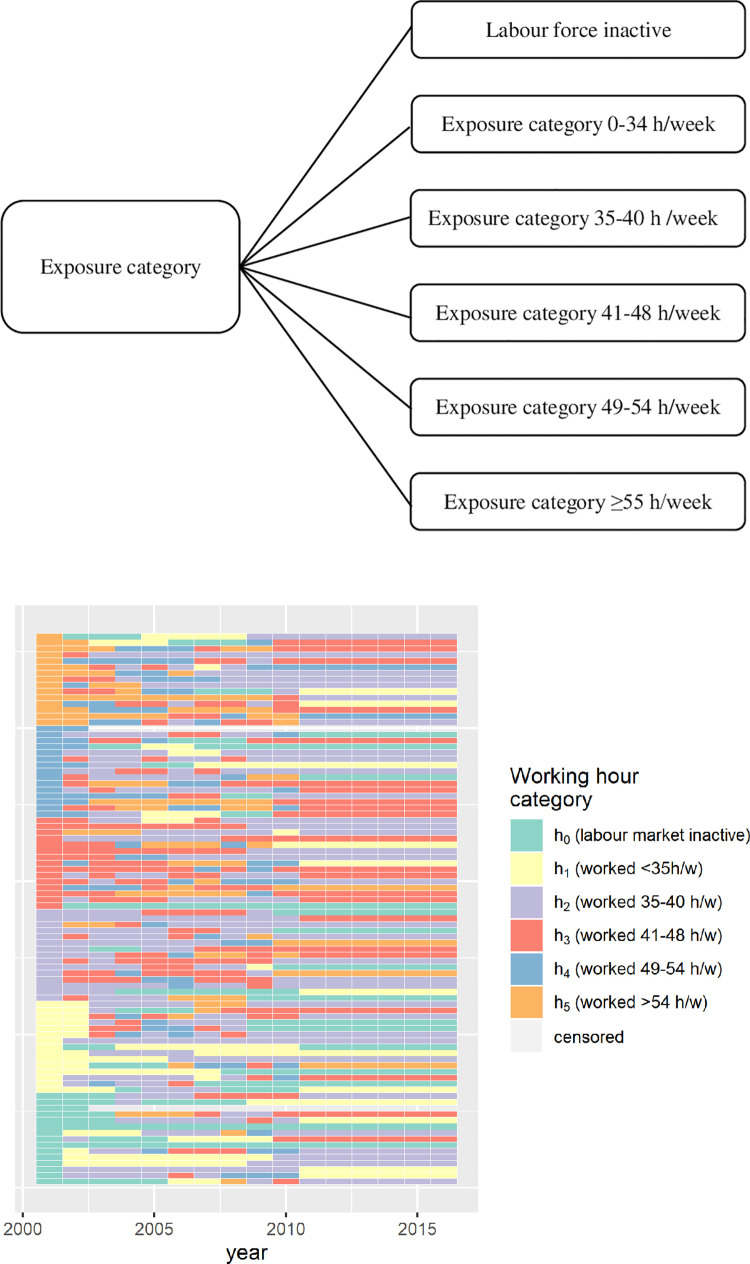
**a) Monte Carlo method based probabilistic model for transitions of a synthetic individual.** The possible transitions from an exposure category at time *year*_*t*_ were into exposure categories labour force inactive, 0–34 hours/week, 35–40 hours/week, 41–48 hours/week, 49–54 hours/week and ≥55 hours/week, and death at time *year*_*t*+1_, with the transition probabilities p_0_ to p_5_ respectively. **b) Evolution of the working hours history of selected 40 years olds (in 2006) Italian males from the Monte Carlo Method generated synthetic cohort**. Each horizontal line represents an individual. The working hour category and termination of follow-up due to death state is coded by the colour.

Model 3, a microsimulation model, was used to estimate the exposed population over the time window in three steps: (a) estimates of prevalence for exposure categories for the first year in the time window (Input Data 1 produced in Model 1); (b) transition probabilities for exposure categories over the entire time window (Input Data 2 produced in Model 2); and (c) number of persons by sex and age at first year of time window, accounting for deaths during the time window. Estimates were derived for the 22 cohorts defined above. The synthetic cohort size was set in the model to follow the age and sex distribution in 2002 and to sum to a total count of *n* = 200,000 to reduce the inherent random noise of MCM. At the time window’s first year, the proportions in exposure categories were initialized to match the point estimates of Model 1 for that year. Transition probabilities obtained from Model 2 were selected according to the actual age of the cohort as it moved through the time window. Termination of follow-up due to death was updated each year according to year, age and sex of the cohort. Part A of Fig 3 shows the possible transitions of a synthetic individual from *year*_*t*_ to *year*_*t*+1_.

The model selection was done by "*fp*" as programmed in STATA 14. The default set from which combinations of powers were tested against each other is m_1,2∈{-2,-1,-0.5,0,0.5,1,2,3} 0 signifies ln(x). Repeated powers imply an additional multiplication of ln(x). Thus, the polynomial with m_1 = 3 and m_2 = 3 implies in our example that age is modelled as x^3+β(x^3 ln(x)), while m_1 = 0 and m_2 = -1 implies βx^(-1)+β(ln(x)). The model with the lowest deviance was selected.

We calculated 95% uncertainty ranges (URs) using bootstrapping [43]. Estimates were produced 1000 times with starting parameters sampled independently from normal distributions with the mean equal to the corresponding point estimate and URs taken from those of the prevalence estimates per year. The 50%, 2.5% and 97.5% quantiles of the resulting random deviates of the exposures were then calculated and assigned to provide the point estimate, and the lower and upper limits of the 95% URs respectively.

## Results

### Prevalence of exposure to long working hours, 2002 (Model 1 output)

In both sexes, 0.5 to 9.0% people were exposed to working hours category of 41–48 h/week, 0.2 to 4.6% were exposed to 49–54 h/week, and 0.2 to 3.4% were exposed to ≥55 h/week across age groups. The corresponding figures for females were 0.2 to 4.4%, 0.1 to 1.7% and 0.1 to 1.3%, and for males 1.0 to 13.6%, 0.3 to 7.5% and 0.3 to 5.6%, respectively (see S4 Table for full breakdown by sex and age group). In general, middle-aged men had higher prevalence of long working hours.

### Transition probabilities over the time window (Model 2 output)

The transition probabilities (Table 1) show that the largest probability is to remain in the initial working hours bands. The most mobile working hours band is *h*_4_ (worked 49–54 hours/week), where strong out movement is present towards both shorter and longer working hours. The least mobile band is *h*_0_ (labour market inactive), because in the youngest (15–19) and oldest (65+) cohorts the largest probability transition is *h*_00_; which is expected as the student or pensioner contributions are significant, respectively.

**Table 1:** Output from Model 2: Transition probability in percentage (and 95% uncertainty intervals) between exposure categories for males and females combined (both sexes) and separately.

Sex	Exposure category at time_t_	Exposure category at time_t+1_, 95% UR					
		*h*_0_ (Labour market inactive or unemployed)	*h*_1_ (worked 0–34 h/w)	*h*_2_ (worked 35–40 h/w)	*h*_3_ (worked 41–48 h/w)	*h*_4_ (worked 49–54 h/w)	*h*_5_ (worked ≥55 h/w)
**Both sexes**	*h*_0_ (Labour market inactive or unemployed)	65.8 (59.2–72.3)	8.9 (8.0–10.0)	11.1 (10.0–12.2)	7.6 (6.8–8.31)	3.2 (2.8–3.5)	5.1 (4.6–5.6)
	*h*_1_ (worked <35 h/w)	29.5 (26.5–32.4)	41.3 (37.1–45.4)	15.8 (14.2–17.3)	8.1 (7.3–9.0)	3.2 (2.9–3.5)	5.2 (4.7–5.8)
	*h*_2_ (worked 35–40 h/w)	23.4 (21.1–25.8)	9.2 (8.3–10.2)	48.9 (44.1–53.8)	11.6 (10.5–12.8)	4.0 (3.6–4.4)	5.7 (5.1–6.2)
	*h*_3_ (worked 41–48 h/w)	23.0 (20.7–25.3)	8.6 (7.7–9.4)	22.6 (20.4–24.9)	37.2 (33.4–40.9)	6.9 (6.2–7.6)	8.3 (7.5–9.1)
	*h*_4_ (worked 49–54 h/w)	24.1 (21.7–26.6)	9.6 (8.7–10.6)	19.8 (17.8–21.8)	17.3 (15.6–19.1)	28.0 (25.2–30.8)	14.3 (12.8–15.7)
	*h*_5_ (worked ≥55 h/w)	24.3 (21.9–26.8)	8.5 (7.7–9.4)	15.7 (14.2–17.3)	13.0 (11.7–14.3)	10.1 (9.1–11.1)	36.9 (33.2–40.6)
**Female**	*h*_0_ (Labour market inactive or unemployed)	70.8 (63.8–77.8)	9.6 (8.7–10.6)	9.3 (8.4–10.2)	5.8 (5.2–6.4)	2.2 (2.0–2.4)	3.2 (2.9–3.6)
	*h*_1_ (worked <35 h/w)	33.0 (29.7–36.3)	45.1 (40.6–49.6)	13.4 (12.1–14.7)	5.8 (5.2–6.3)	2.2 (2.0–2.4)	3.3 (3.0–3.6)
	*h*_2_ (worked 35–40 h/w)	27.1 (24.4–29.8)	10.8 (9.7–11.9)	48.4 (43.6–53.3)	10.3 (9.2–11.3)	3.0 (2.7–3.3)	4.5 (4.0–4.9)
	*h*_3_ (worked 41–48 h/w)	26.4 (23.8–29.1)	10.7 (9.6–11.8)	22.9 (20.6–25.2)	36.3 (32.7–40.0)	6.1 (5.5–6.7)	7.3 (6.6–8.0)
	*h*_4_ (worked 49–54 h/w)	29.0 (26.1–31.9)	12.1 (10.9–13.3)	20.3 (18.3–22.2)	17.2 (15.5–18.9)	27.2 (24.5–29.9)	13.6 (12.2–14.9)
	*h*_5_ (worked ≥55 h/w)	29.5 (26.6–32.5)	10.5 (9.4–11.5)	16.5 (14.8–18.1)	12.4 (11.1–13.6)	9.6 (8.7–10.6)	34.3 (30.7–37.5)
**Male**	*h*_0_ (Labour market inactive or unemployed)	60.5 (54.4–66.5)	8.1 (7.3–8.9)	13.0 (11.7–14.3)	9.4 (8.4–10.3)	4.2 (3.8–4.6)	7.0 (6.3–7.7)
	*h*_1_ (worked <35 h/w)	25.7 (23.2–28.3)	37.2 (33.5–41.0)	18.2 (16.4–20.1)	10.6 (9.6–11.7)	4.3 (3.8–4.7)	7.3 (6.6–8.0)
	*h*_2_ (worked 35–40 h/w)	19.6 (17.6–21.6)	7.6 (6.9–8.4)	49.5 (44.5–54.4)	13.1 (11.8–14.4)	5.1 (4.6–5.6)	6.9 (6.2–7.6)
	*h*_3_ (worked 41–48 h/w)	19.5 (17.5–21.4)	6.3 (5.7–6.9)	22.4 (20.1–24.6)	38.0 (34.2–41.8)	7.8 (7.0–8.6)	9.4 (8.5–10.4)
	*h*_4_ (worked 49–54 h/w)	19.0 (17.1–20.9)	7.1 (6.4–7.8)	19.3 (17.4–21.2)	17.4 (15.7–19.2)	28.8 (25.9–31.6)	15.0 (13.5–16.5)
	*h*_5_ (worked ≥55 h/w)	18.9 (17.0–20.8)	6.5 (5.8–7.1)	15.0 (13.5–16.5)	13.6 (12.3–15.0)	10.6 (9.5–11.6)	39.8 (35.8–43.8)

### Prevalence of exposed population per year in time window (Model 3 output)

A small fraction of a synthetic cohort generated by Model 3 is shown in Part B of Fig 3. Each horizontal line represents the working hour state of a 40-year old (first year of time window) male as a function of time. The working hour category and censoring (due to death) are color-coded. The model structure (i.e., individual changes in working hours in the time window are shown between 2002–2011 and after that only censoring takes place) is clearly observable in the figure.

**Fig 4:**
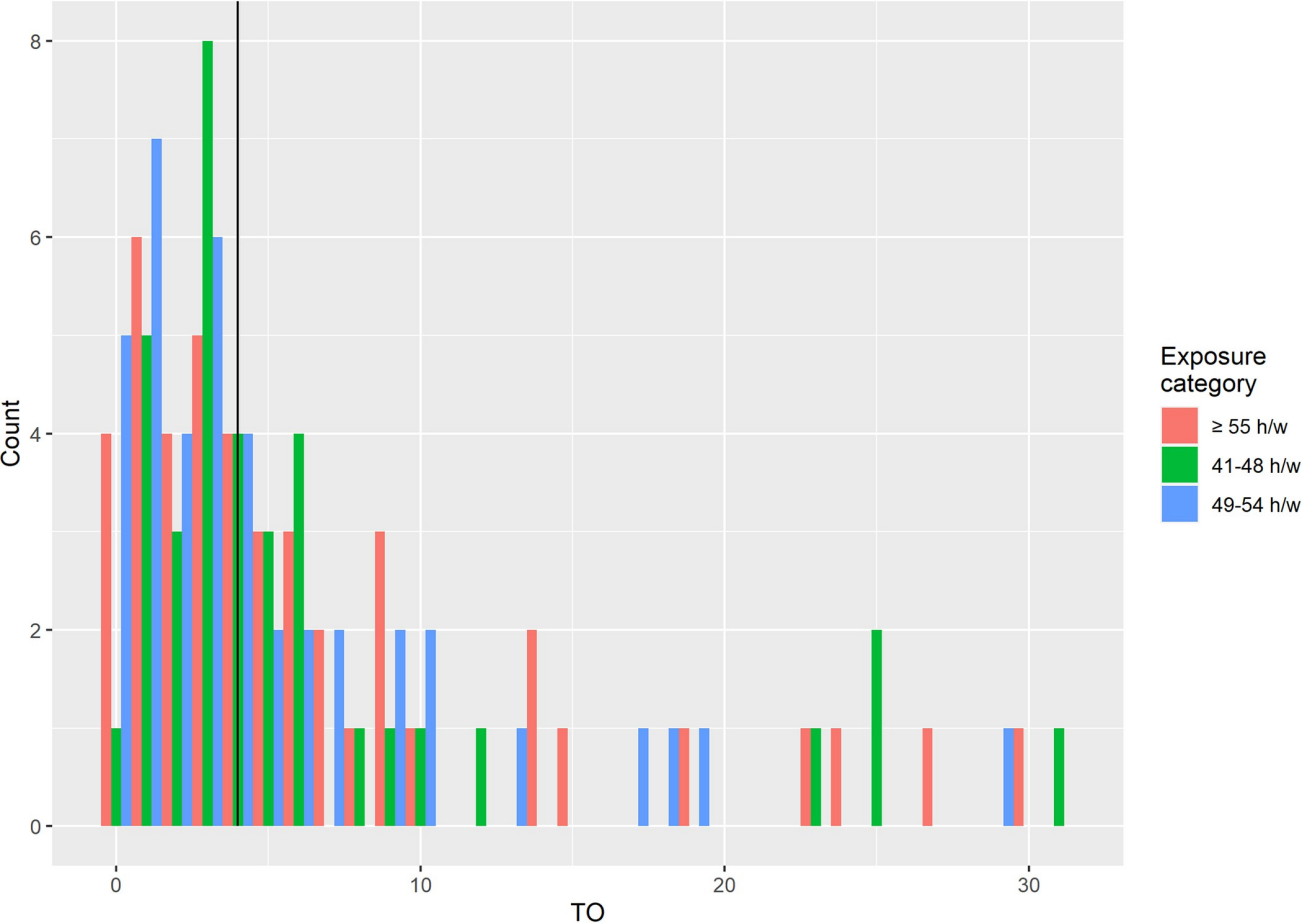
Histogram of the prevalence of exposure to ≥55 h/w in 2016 relative to the prevalence of exposure to ≥55 h/w over the period of 2002–2011. Vertical line at 4 indicates the generally accepted *OT* = 4 for short latency diseases. It is important to note that a single multiplication factor cannot describe all cohorts and exposure categories simultaneously.

From the full set of the generated synthetic cohorts, prevalence of exposures were defined. Fig 4 shows the prevalence of exposure to at each exposure category in 2016 relative to the prevalence of exposure to at each respective category over the period of 2002–2011. In both sexes, the prevalence of exposure per year varies from 0 to 16.5%, 0 to 14.5%, and 0 to 15.7% for working hours categories 41–48, 49–54, and ≥55 h/week, respectively. The corresponding figures for females were 0 to 11.8%, 0 to 8.1% and 0 to 7.2%, and for males 0 to 21.3, 0 to 21.1 and 0 to 24.9% (see S5 Table for a full breakdown by sex and age-group). In general, middle-aged men had higher prevalences.

### Assessing model sensitivity

The sensitivity of the modelling approach was evaluated by calculating median relative error estimates for various input data uncertainty terms and their combinations using tornado plots. Compared to deterministic approaches, the MCM inherently produces random fluctuations of estimates due to its stochastic nature. This numerical noise is solely determined by the size of the synthetic cohort chosen by the modeler. Accordingly, it can be virtually eliminated by an appropriately large synthetic cohort. The fluctuation of the MCM exposure outcomes scale is $\sqrt[{-2}]{n}$ where *n* is the size of the synthetic cohort, as is expected from the law of large numbers (S1A Fig). This observed scaling verifies that the MCM method is numerically robust and there is no loss of power. The URs of the final (“period”) prevalence estimates depend on the URs of the input datasets. To quantify dependence, we repeated the MCM calculations assuming all possible combinations of input data uncertainty. The median relative fluctuation of exposure estimates is presented in S1A Fig. The comparison of the relative importance of variables are presented in terms of relative magnitude of the estimated variance of the estimate (S1B Fig). The highest uncertainty was observed for the working hours data and the working hours transitions (median relative error 5%), followed by working hours data and working hours transitions and WHO life table (<5%). The least uncertainty was for the WHO life table (median relative error <0.05%).

## Discussion

This paper describes a novel computational model for estimating exposure to a risk factor over a time window and demonstrates its application for producing official estimates of exposure to long working hours for a work-related burden of disease study (i.e., the WHO/ILO Joint Estimates). This microsimulation-based method has a number of advantages over other methods. Microsimulation models the individuals in the population and is flexible in how the results can be aggregated. It allows assessment of variations in impact with respect to socio-demographic and geographical parameters. Other applications demonstrating the flexibility of the MCMs in burden of disease estimation are, for example, the DYNAMO-HIA model [44] with which exposure to a risk factor is back-calculated from a known burden of disease (i.e., the inverse process to the method we present here) or the ATHLOS-Mic model [45] with which transition probabilities are estimated used MCMs.

The Global Burden of Disease Study [29] and the United Kingdom Burden of Cancer Study [33,34] used constant values to estimate prevalence of exposure over a time window. It is important to note, however, that estimates derived using different methods cannot be made equal with a single multiplication factor, *OT*, for all exposure categories and cohorts simultaneously (Fig 4). This is particularly important when specific age groups carry proportionally large burden of disease, for example the case for 55 year and older males in the recent WHO/ILO Joint Estimates of the Work-related Burden of Disease and Injury [24]. The *OT* approach loses the accuracy of different exposure categories and is applicable only to binary exposure models. In principle, it is possible to define different *OT*s for different exposure categories and, by that, effectively model different exposure categories independently. The MCM method, on the other hand, can handle arbitrary complex exposure category relations without increasing the model’s complexity. We find that the OT was equal to 4.8 for the entire working-age population (i.e., aged 15 years and older in 2016) exposed to working ≥55 hours/week, whereas previous methods used OTs of 4 [29–32] and 6 [33,34], respectively. Previous methods that used an OT equal to 4, for example, would have underestimated the exposed population by more than 90,000 persons. Moreover, the age- and sex-disaggregated OTs, as estimated using our novel method, vary from 0 to about 20. This indicates a significant gain in precision for estimates disaggregated by age and sex, and that a single scalar multiplicative factor is less favorable.

### Strength and weaknesses

The MCM microsimulation method is a versatile, flexible framework to perform arbitrary complex exposure estimation. It is possible to define exposure according to several different criteria: as described in detail here, it can be defined by the highest exposure category, but other possibilities include the highest exposure category in which at least two consecutive years were spent, or the highest exposure category in which the most time was spent. The model is also useful to produce cumulative exposure estimates. Using input data on occupational risk factor exposures from Occupational Safety and Health modules of labour force surveys, for example, this method provides the potential to estimate exposure to various such risk factors. However, it is limited by the quality of the input data and availability of longitudinal data. If longitudinal labour force surveys are unavailable for a country, data from other countries with a similar economic and social structure could be used as a proxy (with the limitations of this acknowledged). The use of the probabilistic Monte Carlo method for the microsimulation in Model 3 is likely less efficient than use of a deterministic method. The model cannot be applied to continuous exposure data. Also, our modelling assumes the same probability of transitioning from present state to the next state (i.e., exposure in year_t_ was purely calculated based on exposure in year_t-1_), independent of previous states (i.e., the entire exposure regimen, such as exposure in year _t_ could be influenced by exposure in year_t-1_, year_t-2_, etc), which could lead to an overestimate or an underestimate or have no impact on the estimates. It was not possible to model the entire exposure regimen with the data available to us, as the EU-LFS uses a rotating panel design, so individuals are not followed continuously over a period of time. Future research would be needed to test the assumption that transitions are independent of exposure history and, if proven false, microsimulation would need to be developed that takes into consideration full exposure regimen. To improve the approach by overcoming the stochastic nature of the Monte Carlo based microsimulation method, a logical next step is changing Model 3 to a deterministic microsimulation method, such as modelling of microflows [46,47], which we expect to provide gains in model efficiency.

### Application of the estimates of exposed population

To produce occupational burden of disease estimates, accurate estimates are required of the proportion of the population who are exposed to occupational risk factors. Our method has potential to increase accuracy in occupational burden of disease studies. It has already been applied to produce WHO/ILO Joint Estimates of the Work-related Burden of Disease and Injury [21,24]. The models could also be used for other risk factors (e.g., environmental risk factors, such as air pollution, water sanitation and hygiene, and chemicals) and with some modifications to model cumulative exposure, such as cumulative dose (in units of, for example, fibres/m^3^ or mg/m^3^) of occupational exposure to asbestos and silica.

### Conclusion

As part of the WHO/ILO Joint Estimates, we developed a new approach for modelling populations exposed over a time window using detailed transitions probabilities from longitudinal surveys as input data produced by official statistical offices. The method is a versatile and flexible method with good performance. This approach adds accuracy in the modelling of such exposed population numbers and can help advance estimates of the work-related burden of disease. To improve this modelling approach further, we propose a deterministic microsimulation method is next pursued, such as microsimulation of flows.

## Supporting information

S1 Fig **a.** Median relative fluctuation of exposure estimates as a function of the inverse square root of the synthetic cohort population (symbols). Line is a scaling-law expected from the law of large numbers. **b.** The median relative error of exposure estimates (%) due to various input data uncertainty terms and their combination.
(DOCX)

S1 Table Input variables by steps of the estimation and data sources. (DOCX)

S2 Table Metadata on the quarterly EU Labour Force Survey Italy. (DOCX)

S3 Table R code of the multilevel model. (DOCX)

S4 Table Output from Model 1: Estimated prevalence of exposure to working hour catergories with 95% URs in 2002, Italy. (DOCX)

S5 Table Output from Model 3: Estimated prevalence of exposure to working hour catergories with 95% UR over the period of 2002–2011, Italy. (DOCX)

S1 Box Concepts and definitions on temporal relations from occupational burden of disease studies. (DOCX)

S2 Box Modelling approaches used in previous studies. (DOCX)
