# Supplementary material for: Estimating the population exposed to a risk factor over a time window: A microsimulation modelling approach from the WHO/ILO Joint Estimates of the Work-related Burden of Disease and Injury
Source: PLoS One. 2022 Dec 30;17(12):e0278507. doi: 10.1371/journal.pone.0278507 (PMC9803131; doi:10.1371/journal.pone.0278507)
Supplement: S1 Fig — a. Median relative fluctuation of exposure estimates as a function of the inverse square root of the synthetic cohort population (symbols). Line is a scaling-law expected from the law of large numbers. b. The median relative error of exposure estimates (%) due to various input data uncertainty terms and their combination. (DOCX) [file pone.0278507.s001.docx]

| **a**  **b**  **a**  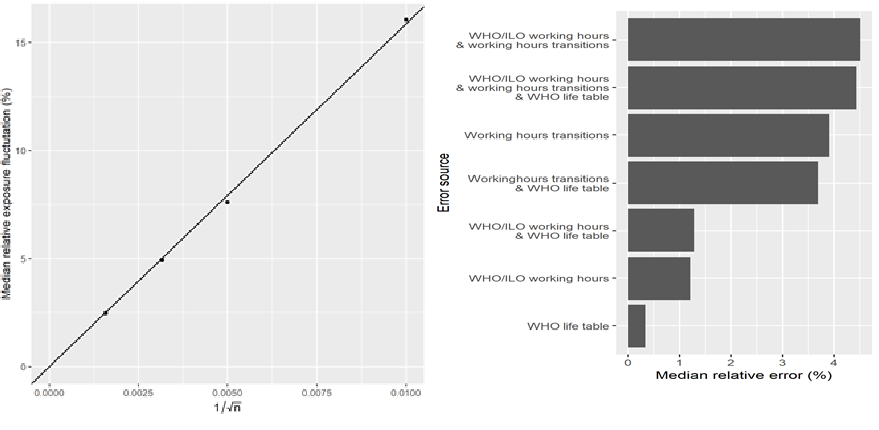 |
| --- |
| **Figure S1a**: Median relative fluctuation of exposure estimates as a function of the inverse square root of the synthetic cohort population (symbols). Line is a scaling-law expected from the law of large numbers.  **S1b:** The median relative error of exposure estimates (%) due to various input data uncertainty terms and their combinations. |
