# Supplementary material for: Estimating the population exposed to a risk factor over a time window: A microsimulation modelling approach from the WHO/ILO Joint Estimates of the Work-related Burden of Disease and Injury
Source: PLoS One. 2022 Dec 30;17(12):e0278507. doi: 10.1371/journal.pone.0278507 (PMC9803131; doi:10.1371/journal.pone.0278507)
Supplement: S1 Table — (DOCX) [file pone.0278507.s002.docx]

**Table S1:** Input variables by steps of the estimation and data sources

| **No** | **Input variable** | **Used in model (Figure 2)** | **Data source** | **Number of surveys (Number of observations used)** |
| --- | --- | --- | --- | --- |
| 1 | Proportion of persons in category of exposure by sex and age at any time unit available | Model 1 | European Union - Labour Force Survey | 2001-2003: sample collected in one week/quarter.  2004-2011: continuous survey  Achieved sample size ca. 250 000 - 300 000 households/500 000 -  600 000 individuals per year reported to Eurostat (repeated interviews) |
| 2 | Proportion of persons in category of exposure by sex and age over the entire time window (i.e. *time_t_* to *time_t+n_*) | Model 2 | European Union - Labour Force Survey | 2001-2003: sample collected in one week/quarter.  2004-2011: continuous survey  Achieved sample size ca. 250 000 - 300 000 households/500 000 -  600 000 individuals per year reported to Eurostat (repeated interviews) |
| 3 | Proportion of persons in category of exposure by sex and age at first year of time window | Model 3 | WHO/ILO Joint Estimates | Output from Model 1 |
| 4 | Average annual transition probabilities between all relevant categories of exposure by sex and age | Output from Model 3 | Experimental estimates based on longitudinal component of EU-LFS | Output from Model 2 |
| 5 | Number of deaths by sex and age at each time unit over time window | Model 3 | WHO life tables | Mortality records from 194 countries reported to WHO^25^ |
| 6 | Number of persons by sex and age at *time_t_* (Figure 1 for nomenclatures) | Model 3 | UN Population Prospects^19^ |  |
