# Supplementary material for: Estimating the population exposed to a risk factor over a time window: A microsimulation modelling approach from the WHO/ILO Joint Estimates of the Work-related Burden of Disease and Injury
Source: PLoS One. 2022 Dec 30;17(12):e0278507. doi: 10.1371/journal.pone.0278507 (PMC9803131; doi:10.1371/journal.pone.0278507)
Supplement: S2 Table — (DOCX) [file pone.0278507.s003.docx]

**Table S2:** Metadata on the quarterly EU Labour Force Survey Italy

| **Covered country** | **Italy** |
| --- | --- |
| Producer of the survey | ISTAT - Istituto Nazionale di Statistica |
| Name of the survey | EU-LFS: European Union Labour Force Survey |
| Covered time period | 1983 to 1996; 1997Q2-ongoing |
| Data collection frequency | annual (spring quarter) 1983-1996; quarterly since 1997q2; from 1983 to 2003 one week per quarter; continuous since 2004 |
| Total number of participants | Achieved sample size of ca. 250 000 to 300 000 households/year. |
| Further relevant metadata on EU-LFS: | https://ec.europa.eu/eurostat/statistics-explained/index.php?title=EU_labour_force_survey |
