# Supplementary material for: Estimating the population exposed to a risk factor over a time window: A microsimulation modelling approach from the WHO/ILO Joint Estimates of the Work-related Burden of Disease and Injury
Source: PLoS One. 2022 Dec 30;17(12):e0278507. doi: 10.1371/journal.pone.0278507 (PMC9803131; doi:10.1371/journal.pone.0278507)
Supplement: S3 Table — (DOCX) [file pone.0278507.s004.docx]

**Table S3:** R code of the multilevel model

my_fit_hours <- lme4::lmer(prop ~ y * poly(a,5) + ((y * poly(a,5)) | country_abbrev),
 data = df,
 weights = wgt)
preds <- merTools::predictInterval(lmerfit_1,
 newdata = df,
 which = "full",
 n.sims = 1000,
 include.resid.var = FALSE,
 level=0.95,
 stat="median")
out_df <- bind_cols(df, preds) %>%
 mutate(fit = case_when(fit < 0 ~ 0,
 fit > 1 ~ 1,
 TRUE ~ fit),
 lwr = case_when(lwr < 0 ~ 0,
 lwr > 1 ~ 1,
 TRUE ~ lwr),
 upr = case_when(upr < 0 ~ 0,
 upr > 1 ~ 1,
 TRUE ~ upr))
