# Supplementary material for: Estimating the population exposed to a risk factor over a time window: A microsimulation modelling approach from the WHO/ILO Joint Estimates of the Work-related Burden of Disease and Injury
Source: PLoS One. 2022 Dec 30;17(12):e0278507. doi: 10.1371/journal.pone.0278507 (PMC9803131; doi:10.1371/journal.pone.0278507)
Supplement: S4 Table — (DOCX) [file pone.0278507.s005.docx]

**Table S4.** **Output from Model 1: Estimated prevalence of exposure to working hour categories with 95% URs in 2002, Italy**

| **Sex** | **Age band** | **Labour market inactive or unemployed** | **95% UR** | **0-34 h/w** | **95% UR** | **35-40 h/w** | **95% UR** | **41-48 h/w** | **95% UR** | **49-54 h/w** | **95% UR** | **≥ 55 h/w** | **95% UR** |
| --- | --- | --- | --- | --- | --- | --- | --- | --- | --- | --- | --- | --- | --- |
| **Both sexes** | All | 57.1 | 56.7-57.6 | 11.3 | 11-11.5 | 22.2 | 21.9-22.5 | 5.1 | 5-5.2 | 2.5 | 2.4-2.5 | 1.8 | 1.7-1.9 |
|  | 15-19 | 90.5 | 89.1-91.9 | 2 | 1-3 | 5.8 | 4.9-6.8 | 1.2 | 0.9-1.6 | 0.2 | 0.1-0.4 | 0.2 | 0-0.4 |
|  | 20-24 | 63.1 | 61.6-64.6 | 8.9 | 8-9.7 | 21.5 | 20.4-22.6 | 4.5 | 4-5 | 1.3 | 1.1-1.4 | 0.8 | 0.4-1.2 |
|  | 25-29 | 41.9 | 40.2-43.7 | 14.4 | 13.5-15.3 | 32.5 | 31.2-33.8 | 7 | 6.4-7.5 | 2.6 | 2.4-2.8 | 1.7 | 1.2-2.1 |
|  | 30-34 | 28.7 | 26.8-30.5 | 18 | 17.1-18.9 | 38.6 | 37.2-40 | 8.5 | 7.9-9.1 | 3.7 | 3.5-3.9 | 2.5 | 2.1-3 |
|  | 35-39 | 23.6 | 21.6-25.5 | 19.6 | 18.7-20.6 | 40.2 | 38.7-41.7 | 9.1 | 8.5-9.6 | 4.4 | 4.2-4.6 | 3.2 | 2.7-3.6 |
|  | 40-44 | 26 | 24.1-27.9 | 19.3 | 18.4-20.3 | 37.9 | 36.5-39.4 | 8.7 | 8.2-9.3 | 4.6 | 4.4-4.8 | 3.4 | 2.9-3.9 |
|  | 45-49 | 34.5 | 32.7-36.3 | 17.4 | 16.5-18.4 | 32.7 | 31.4-34.1 | 7.7 | 7.2-8.2 | 4.3 | 4.1-4.6 | 3.3 | 2.8-3.7 |
|  | 50-54 | 47 | 45.4-48.6 | 14.4 | 13.5-15.3 | 25.8 | 24.6-26.9 | 6.2 | 5.8-6.7 | 3.7 | 3.5-3.9 | 2.9 | 2.4-3.3 |
|  | 55-59 | 61.5 | 60-62.9 | 10.8 | 10-11.7 | 18.2 | 17.2-19.1 | 4.5 | 4.1-5 | 2.8 | 2.6-3 | 2.2 | 1.8-2.6 |
|  | 60-64 | 75.7 | 74.4-77 | 7.2 | 6.4-8.1 | 10.9 | 10.1-11.7 | 2.9 | 2.5-3.2 | 1.8 | 1.6-2 | 1.5 | 1.1-1.8 |
|  | 65-69 | 87.7 | 86.5-88.9 | 4 | 3.2-4.9 | 5.1 | 4.4-5.7 | 1.5 | 1.1-1.8 | 0.9 | 0.8-1.1 | 0.8 | 0.5-1.1 |
|  | 70-74 | 96.1 | 95.1-97.1 | 1.7 | 1-2.4 | 1.2 | 0.5-1.8 | 0.5 | 0.2-0.8 | 0.3 | 0.2-0.4 | 0.3 | 0.1-0.5 |
|  | 75-79 | 99.3 | 98.9-99.8 | 0.6 | 0.2-1 | 0 | 0-0.1 | 0 | 0-0.2 | 0 | 0-0 | 0 | 0-0.1 |
|  | 80-84 | 99.7 | 99.2-100.2 | 0.3 | 0-0.7 | 0 | 0-0.1 | 0 | 0-0.2 | 0 | 0-0 | 0 | 0-0.1 |
|  | 85-89 | 98.9 | 98.1-99.7 | 0.4 | 0-1 | 0.4 | 0-0.8 | 0.1 | 0-0.4 | 0.1 | 0-0.2 | 0.1 | 0-0.2 |
|  | 90-94 | 98.7 | 98-99.4 | 0.2 | 0-0.7 | 0.6 | 0.2-1 | 0.2 | 0-0.5 | 0.1 | 0.1-0.2 | 0.1 | 0-0.3 |
|  | 95+ | 100 | 99.9-100.1 | 0 | 0-0 | 0 | 0-0 | 0 | 0-0 | 0 | 0-0 | 0 | 0-0.1 |
| **Female** | All | 68.8 | 68.2-69.3 | 13.2 | 12.8-13.5 | 14.1 | 13.7-14.5 | 2.4 | 2.3-2.5 | 0.9 | 0.9-0.9 | 0.7 | 0.6-0.8 |
|  | 15-19 | 93.1 | 91.3-95 | 1.6 | 0.1-3.1 | 4.3 | 3.2-5.3 | 0.8 | 0.4-1.1 | 0.1 | 0-0.2 | 0.1 | 0-0.3 |
|  | 20-24 | 68.8 | 66.8-70.8 | 11.1 | 9.6-12.5 | 16.5 | 15.3-17.8 | 2.7 | 2.2-3.1 | 0.6 | 0.5-0.7 | 0.3 | 0.1-0.6 |
|  | 25-29 | 52 | 49.7-54.3 | 18.5 | 17-20 | 23.9 | 22.2-25.5 | 3.9 | 3.4-4.3 | 1.1 | 1-1.3 | 0.7 | 0.4-1 |
|  | 30-34 | 42.9 | 40.4-45.3 | 23.2 | 21.6-24.7 | 27 | 25.3-28.8 | 4.4 | 3.9-4.9 | 1.5 | 1.3-1.6 | 1 | 0.6-1.4 |
|  | 35-39 | 40.7 | 38.1-43.3 | 25 | 23.4-26.7 | 26.9 | 25-28.8 | 4.5 | 3.9-5 | 1.7 | 1.5-1.9 | 1.2 | 0.9-1.6 |
|  | 40-44 | 44.3 | 41.7-46.9 | 24.3 | 22.6-25.9 | 24.3 | 22.4-26.1 | 4.1 | 3.6-4.7 | 1.7 | 1.5-1.9 | 1.3 | 0.9-1.7 |
|  | 45-49 | 52.2 | 49.7-54.6 | 21.4 | 19.7-23 | 20.1 | 18.4-21.8 | 3.5 | 3-4 | 1.6 | 1.4-1.7 | 1.3 | 0.9-1.6 |
|  | 50-54 | 62.7 | 60.4-64.9 | 17.1 | 15.5-18.6 | 15.2 | 13.7-16.7 | 2.7 | 2.3-3.2 | 1.3 | 1.1-1.4 | 1.1 | 0.8-1.4 |
|  | 55-59 | 73.9 | 72-75.9 | 12.1 | 10.7-13.6 | 10.2 | 9-11.5 | 1.9 | 1.5-2.3 | 0.9 | 0.8-1.1 | 0.8 | 0.5-1.1 |
|  | 60-64 | 84.5 | 82.8-86.2 | 7.3 | 6-8.7 | 5.9 | 4.9-6.8 | 1.2 | 0.9-1.5 | 0.6 | 0.5-0.7 | 0.5 | 0.3-0.8 |
|  | 65-69 | 92.9 | 91.4-94.4 | 3.4 | 2.1-4.7 | 2.5 | 1.8-3.2 | 0.6 | 0.3-0.8 | 0.3 | 0.2-0.4 | 0.3 | 0.1-0.5 |
|  | 70-74 | 98.4 | 97.3-99.5 | 0.8 | 0-1.7 | 0.4 | 0-1 | 0.2 | 0-0.4 | 0.1 | 0-0.2 | 0.1 | 0-0.2 |
|  | 75-79 | 100 | 99.6-100.4 | 0 | 0-0.4 | 0 | 0-0.1 | 0 | 0-0.2 | 0 | 0-0 | 0 | 0-0.1 |
|  | 80-84 | 100 | 99.5-100.5 | 0 | 0-0.4 | 0 | 0-0.1 | 0 | 0-0.2 | 0 | 0-0 | 0 | 0-0.1 |
|  | 85-89 | 99.2 | 98.2-100.1 | 0.3 | 0-1.1 | 0.3 | 0-0.7 | 0.1 | 0-0.4 | 0.1 | 0-0.1 | 0.1 | 0-0.2 |
|  | 90-94 | 99.3 | 98.5-100.1 | 0.2 | 0-0.8 | 0.2 | 0-0.6 | 0.1 | 0-0.4 | 0.1 | 0-0.1 | 0.1 | 0-0.2 |
|  | 95+ | 100 | 99.9-100.1 | 0 | 0-0 | 0 | 0-0 | 0 | 0-0 | 0 | 0-0 | 0 | 0-0 |
| **Male** | All | 44.5 | 43.8-45.2 | 9.2 | 8.9-9.5 | 31 | 30.5-31.5 | 8 | 7.8-8.3 | 4.2 | 4.1-4.3 | 3.1 | 2.9-3.3 |
|  | 15-19 | 88 | 85.9-90.1 | 2.5 | 1.2-3.7 | 7.3 | 5.8-8.8 | 1.7 | 1.1-2.3 | 0.3 | 0.1-0.5 | 0.2 | 0-0.6 |
|  | 20-24 | 57.6 | 55.3-59.9 | 6.8 | 5.8-7.7 | 26.3 | 24.6-28 | 6.3 | 5.4-7.1 | 1.9 | 1.6-2.2 | 1.2 | 0.5-1.9 |
|  | 25-29 | 32.1 | 29.5-34.7 | 10.4 | 9.4-11.3 | 40.9 | 38.9-42.9 | 10 | 9-11 | 4 | 3.6-4.4 | 2.7 | 1.8-3.5 |
|  | 30-34 | 14.6 | 11.8-17.4 | 12.9 | 11.9-13.9 | 50 | 47.8-52.3 | 12.5 | 11.5-13.6 | 5.9 | 5.4-6.3 | 4.1 | 3.2-5 |
|  | 35-39 | 6.5 | 3.7-9.3 | 14.2 | 13.2-15.2 | 53.5 | 51.2-55.7 | 13.6 | 12.6-14.6 | 7.1 | 6.7-7.5 | 5.1 | 4.2-5.9 |
|  | 40-44 | 7.5 | 4.8-10.3 | 14.4 | 13.4-15.3 | 51.7 | 49.5-53.9 | 13.4 | 12.4-14.4 | 7.5 | 7.1-8 | 5.5 | 4.6-6.4 |
|  | 45-49 | 16.4 | 13.8-19 | 13.4 | 12.5-14.4 | 45.6 | 43.6-47.7 | 12 | 11.1-12.9 | 7.2 | 6.7-7.6 | 5.3 | 4.5-6.2 |
|  | 50-54 | 30.9 | 28.6-33.3 | 11.7 | 10.8-12.7 | 36.7 | 35-38.4 | 9.8 | 9-10.7 | 6.1 | 5.7-6.5 | 4.7 | 3.9-5.5 |
|  | 55-59 | 48.4 | 46.3-50.6 | 9.5 | 8.5-10.5 | 26.4 | 25-27.9 | 7.3 | 6.5-8 | 4.7 | 4.3-5.1 | 3.7 | 2.9-4.4 |
|  | 60-64 | 66.2 | 64.3-68.2 | 7.1 | 6.1-8.1 | 16.4 | 15.1-17.7 | 4.7 | 3.9-5.4 | 3.1 | 2.8-3.4 | 2.5 | 1.8-3.2 |
|  | 65-69 | 81.8 | 79.9-83.6 | 4.8 | 3.8-5.8 | 7.9 | 6.7-9.2 | 2.4 | 1.8-3.1 | 1.7 | 1.4-1.9 | 1.4 | 0.8-2 |
|  | 70-74 | 93.1 | 91.3-94.9 | 2.9 | 1.9-3.9 | 2.1 | 0.8-3.3 | 0.8 | 0.2-1.5 | 0.6 | 0.4-0.8 | 0.6 | 0-1.1 |
|  | 75-79 | 98.4 | 97.3-99.4 | 1.5 | 0.6-2.4 | 0 | 0-0.2 | 0 | 0-0.4 | 0 | 0-0.1 | 0.1 | 0-0.3 |
|  | 80-84 | 99.2 | 98.3-100.2 | 0.8 | 0-1.6 | 0 | 0-0.2 | 0 | 0-0.5 | 0 | 0-0.1 | 0 | 0-0.2 |
|  | 85-89 | 98.3 | 97-99.7 | 0.5 | 0-1.1 | 0.6 | 0-1.5 | 0.2 | 0-1 | 0.2 | 0-0.4 | 0.1 | 0-0.4 |
|  | 90-94 | 97.1 | 95.6-98.5 | 0.4 | 0-0.9 | 1.5 | 0.4-2.5 | 0.5 | 0-1.2 | 0.3 | 0.1-0.5 | 0.3 | 0-0.6 |
|  | 95+ | 100 | 99.7-100.2 | 0 | 0-0.2 | 0 | 0-0 | 0 | 0-0 | 0 | 0-0 | 0 | 0-0.2 |
