# Supplementary material for: Estimating the population exposed to a risk factor over a time window: A microsimulation modelling approach from the WHO/ILO Joint Estimates of the Work-related Burden of Disease and Injury
Source: PLoS One. 2022 Dec 30;17(12):e0278507. doi: 10.1371/journal.pone.0278507 (PMC9803131; doi:10.1371/journal.pone.0278507)
Supplement: S5 Table — (DOCX) [file pone.0278507.s006.docx]

**Table S5:** Output from Model 3: Estimated prevalence of exposure to working hour categories with 95% CIs over the period of 2002-2011, Italy

| **Sex** | **Age band** | **Labour market inactive or unemployed** | **95% CI** | **0-34 h/w** | **95% CI** | **35-40 h/w** | **95% CI** | **41-48 h/w** | **95% CI** | **49-54 h/w** | **95% CI** | **≥55 h/w** | **95% CI** |
| --- | --- | --- | --- | --- | --- | --- | --- | --- | --- | --- | --- | --- | --- |
| **Both sexes** | All | 31.7 | 31.6-31.8 | 10.2 | 10.1-10.3 | 29.4 | 29.3-29.5 | 8.5 | 8.5-8.6 | 8.9 | 8.9-9 | 9.4 | 9.3-9.5 |
|  | 15-19 | 100 | 100-100 | 0 | 0-0 | 0 | 0-0 | 0 | 0-0 | 0 | 0-0 | 0 | 0-0 |
|  | 20-24 | 87.6 | 86.9-88.4 | 3.6 | 3.2-4.1 | 6.9 | 6.4-7.4 | 1 | 0.9-1.2 | 0.4 | 0.4-0.5 | 0.3 | 0.3-0.4 |
|  | 25-29 | 62.7 | 62.1-63.3 | 10 | 9.7-10.4 | 19.8 | 19.4-20.1 | 3.9 | 3.7-4.1 | 2 | 1.9-2.1 | 1.6 | 1.5-1.7 |
|  | 30-34 | 32.6 | 32.2-33 | 15.9 | 15.7-16.2 | 35 | 34.7-35.4 | 7.8 | 7.6-8 | 4.7 | 4.6-4.9 | 3.9 | 3.8-4.1 |
|  | 35-39 | 10.7 | 10.5-11 | 13.5 | 13.2-13.8 | 44.3 | 43.9-44.8 | 12.6 | 12.4-12.8 | 10 | 9.8-10.2 | 8.8 | 8.5-9.2 |
|  | 40-44 | 6.4 | 6.2-6.6 | 11.5 | 11.2-11.8 | 43.7 | 43.3-44.1 | 13.8 | 13.6-14 | 12.6 | 12.4-12.9 | 12 | 11.5-12.4 |
|  | 45-49 | 5.8 | 5.5-6 | 10.9 | 10.6-11.2 | 41.5 | 41.1-41.9 | 13.6 | 13.4-13.8 | 14 | 13.8-14.3 | 14.1 | 13.7-14.6 |
|  | 50-54 | 7.2 | 6.9-7.6 | 11 | 10.7-11.3 | 38.9 | 38.5-39.3 | 12.8 | 12.6-13 | 14.6 | 14.3-14.8 | 15.5 | 15.1-15.9 |
|  | 55-59 | 11.3 | 10.8-11.8 | 11.4 | 11.1-11.7 | 35.7 | 35.2-36.2 | 11.6 | 11.4-11.8 | 14.2 | 13.9-14.4 | 15.8 | 15.4-16.3 |
|  | 60-64 | 19.6 | 19-20.3 | 11.4 | 11.1-11.7 | 31.5 | 31-32 | 9.8 | 9.6-10 | 12.8 | 12.6-13.1 | 14.9 | 14.4-15.3 |
|  | 65-69 | 33.1 | 32.3-33.9 | 10.7 | 10.4-11.1 | 26.1 | 25.5-26.6 | 7.4 | 7.2-7.6 | 10.3 | 10.1-10.5 | 12.4 | 11.9-12.8 |
|  | 70-74 | 50.7 | 49.8-51.5 | 9.3 | 8.9-9.7 | 19.7 | 19.2-20.3 | 4.7 | 4.6-4.9 | 7 | 6.8-7.2 | 8.6 | 8.2-8.9 |
|  | 75-79 | 68.7 | 67.7-69.6 | 7.3 | 6.8-7.8 | 13.2 | 12.6-13.7 | 2.4 | 2.3-2.5 | 3.8 | 3.6-4 | 4.7 | 4.3-5 |
|  | 80-84 | 83.3 | 82.4-84.3 | 5 | 4.4-5.6 | 7.1 | 6.5-7.6 | 1 | 0.9-1 | 1.6 | 1.5-1.8 | 2 | 1.7-2.3 |
|  | 85-89 | 92.1 | 91.2-92.9 | 3.2 | 2.7-3.8 | 2.9 | 2.4-3.4 | 0.4 | 0.4-0.5 | 0.6 | 0.5-0.7 | 0.8 | 0.6-1 |
|  | 90-94 | 95 | 94.6-95.3 | 2.5 | 2.3-2.7 | 1.6 | 1.5-1.7 | 0.3 | 0.2-0.3 | 0.3 | 0.3-0.3 | 0.4 | 0.3-0.4 |
|  | 95+ | 96.2 | 96-96.5 | 1.8 | 1.6-1.9 | 1.3 | 1.2-1.4 | 0.2 | 0.2-0.2 | 0.2 | 0.2-0.3 | 0.3 | 0.3-0.3 |
| **Female** | All | 40.5 | 40.3-40.7 | 15.6 | 15.4-15.8 | 27.5 | 27.4-27.7 | 5.5 | 5.5-5.6 | 4.6 | 4.6-4.7 | 4 | 3.9-4.1 |
|  | 15-19 | 100 | 100-100 | 0 | 0-0 | 0 | 0-0 | 0 | 0-0 | 0 | 0-0 | 0 | 0-0 |
|  | 20-24 | 91.5 | 90.6-92.3 | 3.5 | 2.9-4.1 | 4.1 | 3.6-4.6 | 0.5 | 0.4-0.7 | 0.2 | 0.1-0.2 | 0.2 | 0.1-0.2 |
|  | 25-29 | 71.8 | 71-72.6 | 11.8 | 11.3-12.4 | 12.9 | 12.5-13.4 | 2 | 1.8-2.2 | 0.8 | 0.8-0.9 | 0.6 | 0.5-0.7 |
|  | 30-34 | 43.8 | 43.2-44.4 | 21.9 | 21.5-22.4 | 26.3 | 25.8-26.7 | 4.3 | 4.1-4.4 | 2.1 | 2-2.2 | 1.6 | 1.5-1.7 |
|  | 35-39 | 17.2 | 16.7-17.7 | 22.2 | 21.7-22.8 | 43.1 | 42.5-43.7 | 8.2 | 7.9-8.4 | 5.3 | 5.1-5.5 | 4 | 3.7-4.3 |
|  | 40-44 | 11 | 10.6-11.4 | 20.2 | 19.7-20.8 | 46.3 | 45.7-46.9 | 9.7 | 9.5-10 | 7.1 | 6.9-7.3 | 5.7 | 5.4-6 |
|  | 45-49 | 10.5 | 10-10.9 | 19.6 | 19.1-20.1 | 45.3 | 44.7-45.9 | 9.9 | 9.6-10.1 | 8.1 | 7.8-8.3 | 6.7 | 6.4-7.1 |
|  | 50-54 | 13.5 | 12.9-14.1 | 19.7 | 19.2-20.3 | 42.1 | 41.4-42.7 | 9.3 | 9-9.5 | 8.2 | 8-8.5 | 7.2 | 6.8-7.6 |
|  | 55-59 | 20.5 | 19.7-21.3 | 19.7 | 19.1-20.3 | 37.1 | 36.4-37.8 | 8.1 | 7.8-8.3 | 7.7 | 7.5-7.9 | 7 | 6.6-7.4 |
|  | 60-64 | 32 | 31-33 | 18.5 | 17.9-19.2 | 30.5 | 29.7-31.3 | 6.4 | 6.2-6.6 | 6.5 | 6.3-6.7 | 6.1 | 5.8-6.5 |
|  | 65-69 | 47.2 | 45.9-48.4 | 16 | 15.4-16.6 | 22.9 | 22.1-23.7 | 4.4 | 4.3-4.6 | 4.8 | 4.6-5 | 4.7 | 4.4-5 |
|  | 70-74 | 63.4 | 62.1-64.7 | 12.5 | 11.7-13.2 | 15.5 | 14.7-16.2 | 2.6 | 2.5-2.7 | 3 | 2.9-3.2 | 3 | 2.7-3.3 |
|  | 75-79 | 78 | 76.6-79.4 | 8.6 | 7.8-9.5 | 9 | 8.3-9.8 | 1.2 | 1.1-1.3 | 1.5 | 1.4-1.7 | 1.6 | 1.3-1.8 |
|  | 80-84 | 88.9 | 87.6-90.1 | 5.1 | 4.2-6 | 4.3 | 3.6-4.9 | 0.5 | 0.4-0.5 | 0.6 | 0.5-0.7 | 0.7 | 0.5-0.9 |
|  | 85-89 | 94.8 | 93.7-95.8 | 2.9 | 2.1-3.6 | 1.6 | 1.2-2.1 | 0.2 | 0.2-0.2 | 0.2 | 0.2-0.3 | 0.3 | 0.2-0.4 |
|  | 90-94 | 96.2 | 95.9-96.5 | 2.3 | 2.1-2.5 | 1.1 | 1-1.1 | 0.2 | 0.1-0.2 | 0.1 | 0.1-0.2 | 0.2 | 0.1-0.2 |
|  | 95+ | 96.9 | 96.7-97.2 | 1.7 | 1.6-1.9 | 0.9 | 0.8-1 | 0.1 | 0.1-0.1 | 0.1 | 0.1-0.2 | 0.2 | 0.1-0.2 |
| **Male** | All | 22.2 | 22-22.3 | 4.4 | 4.3-4.4 | 31.4 | 31.3-31.6 | 11.8 | 11.7-11.9 | 13.6 | 13.5-13.7 | 15.2 | 15-15.4 |
|  | 15-19 | 100 | 100-100 | 0 | 0-0 | 0 | 0-0 | 0 | 0-0 | 0 | 0-0 | 0 | 0-0 |
|  | 20-24 | 84 | 82.8-85.2 | 3.8 | 3.2-4.4 | 9.5 | 8.7-10.4 | 1.5 | 1.3-1.7 | 0.7 | 0.6-0.8 | 0.5 | 0.4-0.6 |
|  | 25-29 | 53.9 | 53.1-54.7 | 8.3 | 8-8.7 | 26.4 | 25.8-27 | 5.8 | 5.5-6.1 | 3.2 | 3-3.3 | 2.5 | 2.3-2.7 |
|  | 30-34 | 21.5 | 21.1-22 | 10 | 9.7-10.2 | 43.7 | 43.2-44.2 | 11.2 | 10.9-11.6 | 7.3 | 7.1-7.6 | 6.2 | 5.9-6.5 |
|  | 35-39 | 4.3 | 4.1-4.5 | 4.8 | 4.6-4.9 | 45.6 | 45-46.2 | 17 | 16.6-17.4 | 14.7 | 14.3-15.1 | 13.7 | 12.9-14.4 |
|  | 40-44 | 1.7 | 1.6-1.8 | 2.7 | 2.6-2.9 | 41.1 | 40.6-41.7 | 17.9 | 17.6-18.2 | 18.2 | 17.8-18.6 | 18.3 | 17.5-19.1 |
|  | 45-49 | 1 | 0.8-1.1 | 2 | 1.8-2.1 | 37.7 | 37.1-38.3 | 17.4 | 17.1-17.8 | 20.2 | 19.7-20.6 | 21.7 | 20.9-22.5 |
|  | 50-54 | 0.7 | 0.4-1 | 1.9 | 1.7-2.1 | 35.6 | 35-36.1 | 16.6 | 16.2-16.9 | 21.1 | 20.7-21.6 | 24.1 | 23.3-24.8 |
|  | 55-59 | 1.6 | 1.1-2.2 | 2.5 | 2.3-2.8 | 34.2 | 33.7-34.8 | 15.3 | 15-15.7 | 21.1 | 20.6-21.5 | 25.2 | 24.3-26 |
|  | 60-64 | 6.3 | 5.5-7.1 | 3.7 | 3.5-4 | 32.6 | 32-33.2 | 13.5 | 13.2-13.8 | 19.6 | 19.2-20.1 | 24.3 | 23.5-25.1 |
|  | 65-69 | 17.7 | 16.7-18.8 | 4.9 | 4.7-5.2 | 29.5 | 28.9-30.1 | 10.7 | 10.4-11 | 16.3 | 15.9-16.7 | 20.8 | 20-21.6 |
|  | 70-74 | 35.9 | 34.9-37 | 5.6 | 5.3-6 | 24.7 | 24-25.4 | 7.2 | 7-7.5 | 11.6 | 11.2-11.9 | 15 | 14.2-15.7 |
|  | 75-79 | 56.8 | 55.6-58.1 | 5.6 | 5.1-6 | 18.4 | 17.7-19.2 | 4 | 3.7-4.2 | 6.7 | 6.4-7 | 8.6 | 7.8-9.3 |
|  | 80-84 | 75.2 | 73.7-76.6 | 4.9 | 4.3-5.4 | 11.2 | 10.3-12.1 | 1.7 | 1.6-1.9 | 3.1 | 2.9-3.4 | 3.9 | 3.3-4.5 |
|  | 85-89 | 86.9 | 85.4-88.4 | 3.9 | 3.3-4.6 | 5.3 | 4.2-6.4 | 0.8 | 0.7-0.9 | 1.3 | 1.1-1.6 | 1.7 | 1.2-2.2 |
|  | 90-94 | 91.8 | 90.9-92.7 | 3.1 | 2.5-3.7 | 3 | 2.8-3.2 | 0.6 | 0.5-0.6 | 0.7 | 0.6-0.7 | 0.9 | 0.7-1.1 |
|  | 95+ | 93.7 | 93.2-94.2 | 1.9 | 1.6-2.2 | 2.6 | 2.3-2.9 | 0.4 | 0.4-0.5 | 0.6 | 0.5-0.7 | 0.7 | 0.6-0.9 |
