# Supplementary material for: Estimating the population exposed to a risk factor over a time window: A microsimulation modelling approach from the WHO/ILO Joint Estimates of the Work-related Burden of Disease and Injury
Source: PLoS One. 2022 Dec 30;17(12):e0278507. doi: 10.1371/journal.pone.0278507 (PMC9803131; doi:10.1371/journal.pone.0278507)
Supplement: S1 Box — (DOCX) [file pone.0278507.s007.docx]

**Box S1:** Concepts and definitions on temporal relations from occupational burden of disease studies

| **Term** | **Definition** |
| --- | --- |
| Occupational turnover rate | Ratio of the prevalence of a category of exposure to the risk factor at one year divided by the prevalence of the same exposure category over a time window |
| Transition probability between exposure categories | Probability of changing the level of exposure between the exposure categories |
| Time window of exposure to the risk factor | A time period during which exposure to a risk factor could have led to the health outcome in the estimation year |
| Attributable burden | Burden of disease that can be attributed to exposure to a particular risk factor in the past. |
| Lag time | Time period between exposure to the risk factor and the occurrence of the health outcome. |
