# Supplementary material for: Estimating the population exposed to a risk factor over a time window: A microsimulation modelling approach from the WHO/ILO Joint Estimates of the Work-related Burden of Disease and Injury
Source: PLoS One. 2022 Dec 30;17(12):e0278507. doi: 10.1371/journal.pone.0278507 (PMC9803131; doi:10.1371/journal.pone.0278507)
Supplement: S2 Box — (DOCX) [file pone.0278507.s008.docx]

**Box S2:** Modelling approaches used in previous studies

| **Study** | **Modelling approach used** |
| --- | --- |
| *Global Burden of Disease Study* ^11-14^ | The occupational turnover (OT) rate for risk factors:  $OT=\frac{P_{t}}{P_{0}}=\frac{orignal workers+new workers-deaths}{original workers}=\left( 1-m \right)\left( 1+ART*t \right)$  where *ART* is the annual rate of turnover, *m* is the mortality rate and *t* is the total number of years of working life. |
| *United Kingdom Burden of Cancers Study* ^15, 16^ | The number of people ever exposed during the risk exposure period (REP), *N*_e(REP)_, taking into account turnover was calculated as:  $N_{e(REP)}=n_{0}+\{n_{0} \times TO \times t\}$  Where, *n_0_* is the number of people employed at the midpoint of the REP, *TO* is staff turnover per year and *t* is the number of years in the REP. |
